# Effects of support from spouse on child birth among rural Indian women

**DOI:** 10.6026/973206300222843

**Published:** 2026-05-31

**Authors:** Renu Waghmare, Purti Agrawal Saini, Chetan Sharma, Anjali Arya, Ramesh Agrawal

**Affiliations:** 1 Department of Community Medicine, Virendra Kumar Sakhlecha Government Medical College, Neemuch, Madhya Pradesh, India; 2 Department of Pathology, Nandkumar Singh Chouhan Government Medical College, Khandwa, Madhya Pradesh, India; 3 Department of Microbiology, Virendra Kumar Sakhlecha Government Medical College Neemuch, Madhya Pradesh, India; 4 Department of Microbiology, Government Medical College, Satna, Madhya Pradesh, India

**Keywords:** Birth preparedness and complication readiness (BPCR), husband in maternal and newborn health (MNH), maternal mortality rate

## Abstract

Inadequate husband involvement remains a major barrier to effective birth preparedness and complication readiness (BPCR) for maternal and newborn care in rural India. Hence, a cross-sectional study was conducted from June 2025 to December 2025 at the Rural Health and Training Centre, Manglia, including 283 pregnant woman-husband dyads. The BPCR index was 45.86, indicating suboptimal preparedness in the study population. Significant associations were observed between husband involvement and completion of 4 ANC visits (OR=1.7), knowledge of one key danger sign of pregnancy (OR=1.8) and awareness of essential newborn care (OR=1.8). Thus, we show that husbands' participation is significantly linked to better BPCR-related practices, supporting their inclusion in maternal counselling strategies.

## Background:

The Cairo International Conference on Population and Development (ICPD) in 1994 was the first global initiative to emphasize the shared responsibility of men in RMNCH. The program explicitly called on policy-makers and programmers to engage men in RMNCH actions, including promoting their active involvement in parenthood and family planning [1]. More recently, increased involvement of men has been seen in maternal and newborn health (MNH). In 2015, the World Health Organization (WHO) issued a recommendation encouraging interventions to promote male involvement and engagement to increase the utilization of skilled MNH services [2]. In patriarchal societies, pregnancy and childbirth are often regarded as women's exclusive concerns. At the same time men have their own decision-making, which is welcomed in our society. Men play a role in the coordination of various activities that are essential for a child birth as arranging for transport, blood donor, arranging for blood investigations and processes linked to Janani Suraksha Yojana (JSY) which can aid in money grant to the patient [3]. Therefore, playing a crucial role in reducing the 'three delays'(i.e., the delay in deciding to seek care, the delay in reaching health services and the delay in receiving adequate and appropriate treatment once at a health facility), thereby facilitating women's access to and utilization of skilled MNH services [4]. High coverage and quality of antenatal care (ANC), postnatal care (PNC) and skilled attendance at birth are major drivers of improving MNH and averting avoidable maternal and newborn deaths [5]. Therefore, it is of interest to describe the role of male involvement in birth preparedness and complication readiness, as their engagement can significantly improve maternal and newborn health outcomes by addressing the 'three delays' and promoting the utilization of skilled MNH services.

## Materials and Methods:

The present study was undertaken in the field practice area of the Rural Health and Training centre, Mangliya, which is attached to the Department of Community Medicine, Shri Aurobindo Medical College and PG Institute, Indore. The present study is a cross-sectional observational study, carried out for a period of one and half years from June 2025 to December 2025. All adolescent pregnant females and their husbands attending the anganwadi and those seeking care at Rural Health and Training centre, Mangliya and those who gave consent were included in the study. Rural Health and Training Centre, Mangliya is 18 kilometers away from the Institute and is located at the west of Indore district, with a population of 28,176 according to 2011 census. All pregnant women 19-49 years of age and their husbands attending anganwadi and those who visited at rural health and training centre Manglia during the study period were included in the study. All those who provided consent for the study were enrolled in the study rest all were excluded. Women with a history of abortion during the 12 months preceding the survey and their husbands were excluded from the study to avoid any potential psychological trauma to the respondents by recalling the events associated with it. There are 28 anganwadi is in the field practice area of Manglia and all the anganwadi were included in the study. For each anganwadi one day was fixed in a week when all pregnant females with their husbands were requested to be present for their antenatal visit. Anganwadi workers' help was sought to inform all the pregnant females and the purpose of the study was explained and verbal consent was obtained before the interview. This was cross-checked with the line list of pregnant females already made by the anganwadi workers and ASHA and care was taken to enroll a greater number of study participants by conducting extra sessions on fixed immunization days. On these particular days, the study participants were interviewed using a pilot-tested predesigned semi-structured questionnaire that consisted of a sociodemographic profile and validated questionnaire related to birth preparedness and complication readiness. The age of the participant was cross-checked by voter identity card. Study data from 283 wife-husband dyads were obtained from 580 antenatal cases. The recorded data were entered intoan MS Excel sheet. Percentages, Z-test and chi-square were used for statistical analysis. The calculation of the BPCR index was performed considering 13 indicators, where the presence of each indicator was awarded with 1 mark, making the maximum as 13 pointer scale of the BPCR index. Two indicators in the list are important for recently 11 delivered woman; hence, 11 indicators were considered for the calculation. After awarding 1 mark each, it was expressed in percentages and the mean of the percentage was calculated. This is BPCR index. As this is a part of large study hence only male involvement is being focused in the research article.

## Results:

Table 1 presents the sociodemographic profile of the 283 pregnant women and their husbands included in the study. Among the pregnant women, nearly half were in the 15-24 years age group (48.76%), followed by 43.81% in the 25-44 years group, while 7.42% were aged more than 45 years. The mean age of the pregnant women was 22.6 ± 5.2 years, indicating that the study population was predominantly young. With regard to educational status, husbands were generally better educated than pregnant women. Among the women, 28.62% had education ranging from no schooling to primary incomplete, 47.70% had primary complete to secondary incomplete education and only 23.67% had attained secondary education or higher. In contrast, among husbands, 14.84% were in the lowest educational category, 49.46% had primary complete to secondary incomplete education and a relatively higher proportion, 35.68%, had secondary education or above. This pattern is also reflected in the mean years of schooling, which was 6.8 ± 4.2 years for pregnant women compared with 8.9 ± 6.5 years for husbands. These findings suggest an educational advantage among husbands, which may influence awareness, decision-making and participation in maternal care. In terms of social category, the majority of both pregnant women and husbands belonged to the unreserved category, accounting for 82.68% and 80.91%, respectively, while 15.90% of women and 17.66% of husbands belonged to the reserved category. This indicates that most study participants were drawn from socially non-reserved groups. Regarding socioeconomic status, as classified by the modified B.G. Prasad classification, the largest proportion of families belonged to the middle class (39.92%), followed by the lower middle class (30.74%). Smaller proportions were from the upper middle class (18.72%), lower class (5.65%) and upper class (1.76%). Thus, the study population was concentrated mainly in the middle and lower-middle socioeconomic strata, suggesting that the sample largely represented families with moderate socioeconomic resources. Women receiving ANC, skilled care at birth, PNC and care for obstetric complications from trained healthcare providers were considered outcome variables, as shown in (Table 2). More than 50 % females had >4 ANC visits (60.77%), had undergone institutional delivery (69.96%), identified blood donor (57.95%), had knowledge about government transport schemes (68.91%) and more than 80% saved money for pregnancy, identified transport (88.69%) and had knowledge about cash incentives scheme (92.93%). The birth preparedness and complication readiness index is average score of all the variables as answered by pregnant females was found to 45.86. The association was found to be significant at p < 0.05 for four ANC visits (OR= 1.7), knowing one key danger sign of pregnancy (OR=1.8) and knowing the key components of essential newborn care (OR=1.8), as shown in (Table 3). Hence, the value of birth preparedness and complication readiness was better estimated when participation of husbands was evaluated.

## Discussion:

In passing from the era of the Millennium Development Goals (MDG) to the United Nations 2030 Agenda for Sustainable Development, the international community has established the Sustainable Development Goals (SDGs) and set the target for countries to reduce maternal mortality ratio to less than 70 per 100,000 live births by 2030 [6]. Age plays a significant role in predicting birth preparedness, with adult pregnancies demonstrating higher levels of preparedness compared to adolescent pregnancies. The study found that women of adult age, particularly those between 21 and 25 years, exhibited significantly greater knowledge of key danger signs of pregnancy. This finding aligns with the study by Calle (2021) *et al.* [7] where the knowledge of danger signs was not universal, but adult pregnancies showed improved awareness. The odds of birth preparedness and complication readiness were nearly twice as high among women knowledgeable about key danger signs compared to those who were not. In this study, 69.96% of women had institutional deliveries, a rate higher than that reported in the DLHS-3 report for Uttar Dinajpur and comparable to the NFHS-3 report for West Bengal and India. However, limited access to resources such as liquid cash and transport, particularly in remote areas, remains a significant barrier to accessing skilled healthcare. The study showed that nearly 90% of women had saved money or identified a vehicle for emergency transportation, crucial steps in birth preparedness and complication readiness (BPCR). Government schemes like Janani Suraksha Yojana (JSY) and referral transport were noted to facilitate access, particularly for marginalized populations, although awareness of referral transport schemes was poor [8]. Knowledge of danger signs during pregnancy, labor, postpartum and for the newborn ranged from 19% to 48.76% in the study population, reflecting a concerning gap in health communication. The study also found that while identifying a blood donor for obstetric emergencies was not considered essential by most participants, a significant percentage had taken steps to prepare for emergencies through other measures like saving money and arranging transport. The findings were consistent with those of Dangura *et al.* (2020) [9], who highlighted the gaps in danger sign awareness and emphasized the need for better health communication. Interestingly, birth preparedness was better among general category women compared to those from backward classes. However, women from backward castes and below the poverty line (BPL) families were more aware of government cash incentives, potentially explaining this paradox. Educational status was a strong predictor of birth preparedness awareness, with women who had more than five years of education showing higher awareness of BPCR practices [10]. Husband involvement also emerged as a crucial factor in reducing the well-established "three delays" model: delay in deciding to seek care, delay in reaching a health facility and delay in receiving appropriate treatment. Male awareness of obstetric danger signs facilitated early recognition and decision-making, while logistical planning, such as emergency transport and savings, helped reduce delays [11]. As seen in Danna *et al.* study [12], spousal preparedness plays an essential role in improving maternal and neonatal health outcomes by preventing avoidable morbidity and mortality [13].

## Conclusion:

We show that birth preparedness and complication readiness was low, particularly in areas such as saving money, arranging transport and awareness of governmental schemes. Increasing literacy and promoting late-age conception can enhance knowledge of key danger signs and improve preparedness. Involving husbands in antenatal care improves awareness and access to skilled care, which is crucial in reducing maternal and perinatal mortality and morbidity.

## Figures and Tables

**Table 1:** Sociodemographic characteristics of pregnant females and their husbands (Dyads)

**Sociodemographic Variables**		**Pregnant Females**	**Husbands**
		283 n (%)	283
Age	15-24 years	138 (48.76%)	-
	25-44 years	124 (43.81%)	-
	>45 years	21 (7.42%)	-
Mean age in years		22.6 (+5.2) years	-
Education	No education to Primary incomplete	81 (28.62)	42 (14.84)
	Primary complete and secondary incomplete	135 (47.70)	140 (49.46)
	Secondary complete or higher	67 (23.67)	101(35.68)
Mean years of schooling		6.8 (±4.2)	8.9 (±6.5)
Category	Reserved	45 (15.90)	50 (17.66)
	Unreserved	234 (82.68)	229 (80.91)
Socioeconomic class	Upper class	5 (1.76)	5 (1.76)
(according to modified BJ	Upper middle class	53 (18.72)	53 (18.72)
Prasad classification)	Middle class	113 (39.92)	113 (39.92)
	Lower middle class	87 (30.74)	87 (30.74)
	Lower class	16 (5.65)	16 (5.65)

**Table 2:** BPCR variables assessing and husband's knowledge among study participants

**BPCR INDICATORS**	**Yes**	**No**
Registration < 12 weeks	104 (36.74)	179 (63.25)
ANC visits >4	172 (60.77)	111(39.22)
Institutional delivery	198 (69.96)	85 (30.03)
Saving money for Pregnancy	251(88.69)	32 (11.30)
Identifying blood donor	164 (57.95)	119(42.04)
Identifying transport	251 (88.69)	32(11.30)
Knowing 1 key danger sign of pregnancy	138 (48.76)	145(51.23)
Knowing 1 key danger sign of labor	39 (13.78)	244(86.21)
Knowing 1 key danger sign of postpartum	22 (7.7)	261(92.22)
Knowing 1 key danger sign of new born	19 (6.71)	264(93.28)
Knowing 1 key component of essential new born care	42 (14.84)	241(85.15)
Govt. cash incentive Scheme	263 (92.93)	20(7.06)
Govt. transport scheme	195 (68.9)	88(31.9)
(%) percentages are
expressed in parenthesis.

**Table 3:** Association of husband's knowledge regarding BPCR variables under study

**BPCR INDICATORS**	**OR (Cl)**	**AOR (Cl)**
Registration < 12 weeks	1.4(0.8-1.6)	1.3 (0.9-1.6)
ANC visits >4	1.7*(0.9-1.9)	1.7* (1.01–3.0)
Institutional delivery	1.2(0.8-1.4)	1.1(0.8-1.5)
Saving money for Pregnancy	1.2(0.8-1.2)	1.2(0.9-1.3)
Identifying blood donor	1.4(0.6-1.9)	1.3(0.5-1.8)
Identifying transport	1.4(0.8-1.6)	1.4(0.7-1.5)
Knowing 1 key danger sign of pregnancy	1.8*(1.2-1.9)	1.7*(1.2-2.01)
Knowing 1 key danger sign of labor	1.4(1.2-1.8)	1.3(1.2-1.9)
Knowing 1 key danger sign of postpartum	1.2(0.8-1.2)	1.2(0.8-1.2)
Knowing 1 key danger sign of new born	1.2(0.8-1.4)	1.1(0.9-1.3)
Knowing 1 key component of essential new born care	1.8*(1.2-2.2)	1.9*(1.1-2.09)
Govt. cash incentive Scheme	1.5(1.4-2.9)	1.5* (1.09-3.0)
Govt. transport scheme	1.4(0.8-1.7)	1.3(0.9-1.9)
*p <0.05
